# Supplementary material for: Tibial Damage Caused by T-2 Toxin in Goslings: Bone Dysplasia, Poor Bone Quality, Hindered Chondrocyte Differentiation, and Imbalanced Bone Metabolism
Source: Animals (Basel). 2024 Aug 5;14(15):2281. doi: 10.3390/ani14152281 (PMC11311038; doi:10.3390/ani14152281)
Supplement: Supplementary file 1 [file animals-14-02281-s001.zip › Supplementary Figure S1.pdf]

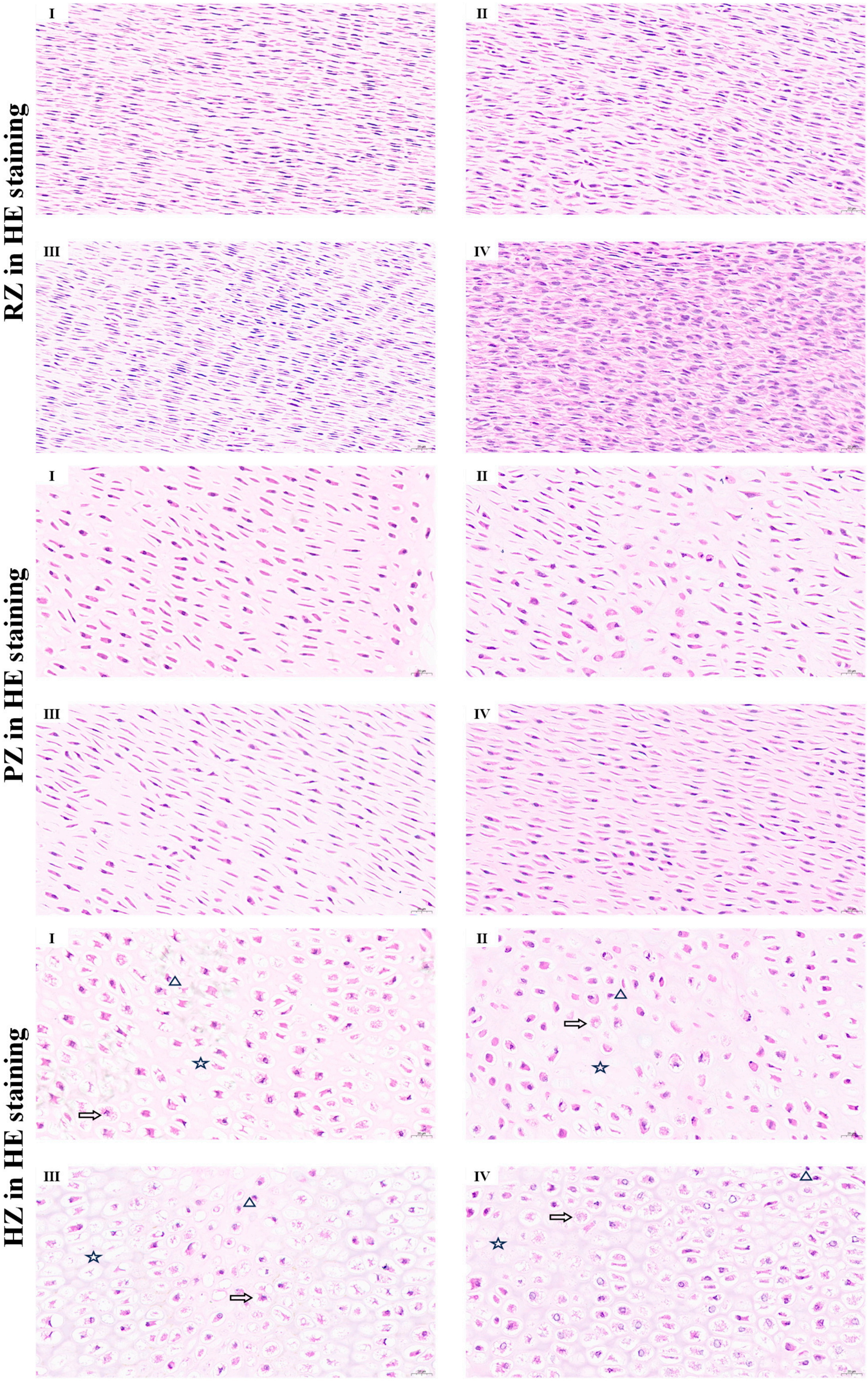
RZ in HE staining
PZ in HE staining
HZ in HE staining
I
II
III
IV

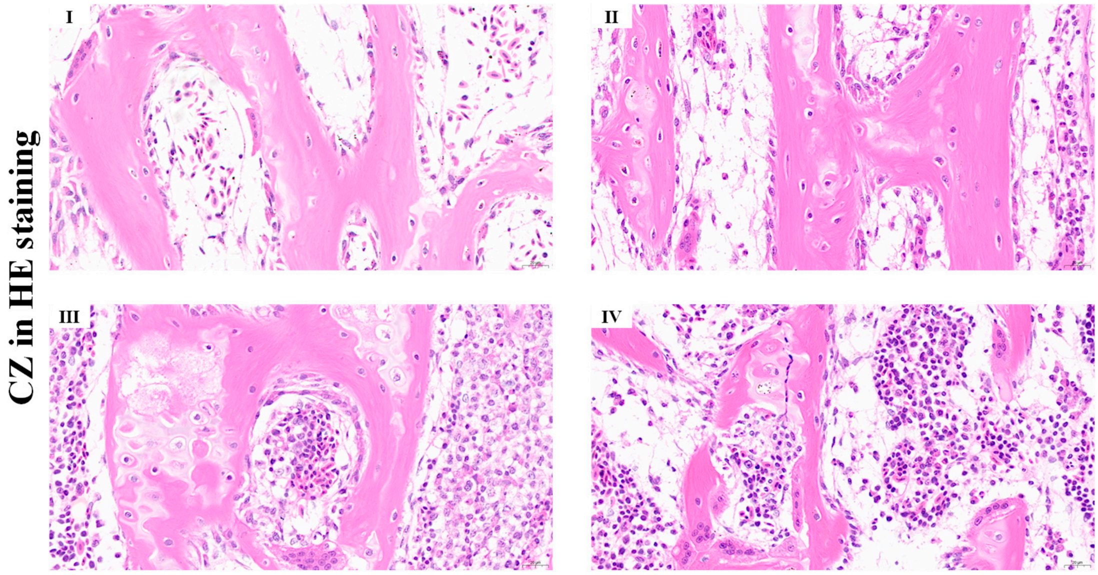

Supplementary Figure S1. The representative images of higher magnifications of TGP in HE staining. Ⅰ, 0 mg/kg T-2 toxin group; Ⅱ, 0.5 mg/kg T-2 toxin group; Ⅲ, 1.0 mg/kg T-2 toxin group; Ⅳ, 2.0 mg/kg T-2 toxin group; HE, hematoxylin-eosin; Cellular vacuolization (black star); Necrosis (black arrow); Apoptosis (black triangle). A scale bar of 100 μm.
