# Supplementary material for: Tibial Damage Caused by T-2 Toxin in Goslings: Bone Dysplasia, Poor Bone Quality, Hindered Chondrocyte Differentiation, and Imbalanced Bone Metabolism
Source: Animals (Basel). 2024 Aug 5;14(15):2281. doi: 10.3390/ani14152281 (PMC11311038; doi:10.3390/ani14152281)
Supplement: Supplementary file 1 [file animals-14-02281-s001.zip › Supplementary Figure S2.pdf]

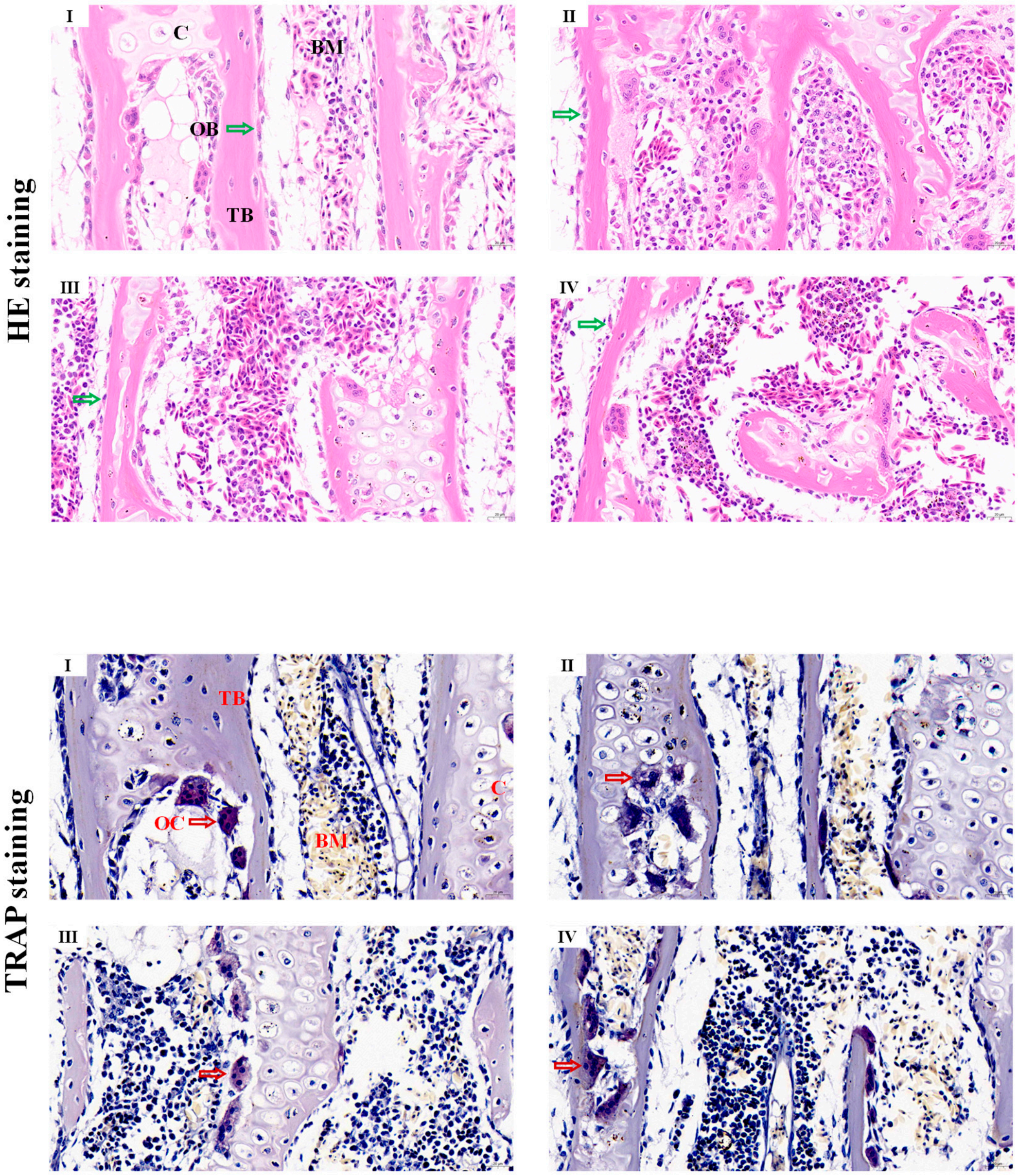

Supplementary Figure S2. The representative images of higher magnifications of osteoblasts and osteoclasts.
